# *Larix laricina*, an Antidiabetic Alternative Treatment from the Cree of Northern Quebec Pharmacopoeia, Decreases Glycemia and Improves Insulin Sensitivity *In Vivo*

**DOI:** 10.1155/2012/296432

**Published:** 2012-07-19

**Authors:** Despina Harbilas, Diane Vallerand, Antoine Brault, Ammar Saleem, John T. Arnason, Lina Musallam, Pierre S. Haddad

**Affiliations:** ^1^Canadian Institutes of Health Research Team in Aboriginal Antidiabetic Medicines, Department of Pharmacology, University of Montreal, P.O. Box 6128, Downtown Station, Montreal, QC, Canada H3C 3J7; ^2^Natural Health Products and Metabolic Diseases Laboratory, Department of Pharmacology, University of Montreal, Montreal, QC, Canada H3C 3J7; ^3^Institute of Nutraceuticals and Functional Foods, Laval University, Quebec City, QC, Canada G1V 0A6; ^4^Montreal Diabetes Research Center, University of Montreal Hospital Center, Montreal, QC, Canada H1W 4A4; ^5^Department of Biology and Center for Research in Biopharmaceuticals and Biotechnology, University of Ottawa, Ottawa, ON, Canada K1N 6N5

## Abstract

*Larix laricina* K. Koch is a medicinal plant belonging to traditional pharmacopoeia of the Cree of Eeyou Istchee (Eastern James Bay area of Canada). *In vitro* screening studies revealed that, like metformin and rosiglitazone, it increases glucose uptake and adipogenesis, activates AMPK, and uncouples mitochondrial function. The objective of this study was to evaluate the antidiabetic and antiobesity potential of *L. laricina* in diet-induced obese (DIO) C57BL/6 mice. Mice were subjected for eight or sixteen weeks to a high fat diet (HFD) or HFD to which *L. laricina* was incorporated at 125 and 250 mg/kg either at onset (prevention study) or in the last 8 of the 16 weeks of administration of the HFD (treatment study). *L. laricina* effectively decreased glycemia levels, improved insulin resistance, and slightly decreased abdominal fat pad and body weights. This occurred in conjunction with increased energy expenditure as demonstrated by elevated skin temperature in the prevention study and improved mitochondrial function and ATP synthesis in the treatment protocol. *L. laricina* is thus a promising alternative and complementary therapeutic approach for the treatment and care of obesity and diabetes among the Cree.

## 1. Introduction

The prevalence of obesity and type 2 diabetes (T2D) has reached epidemic proportions worldwide. In Canada alone, obesity and T2D affect 25% [1–3] and roughly 6% [4] of the population, respectively. The incidence of these diseases is worsened among the aboriginal population of Canada. The Cree of Eeyou Istchee (CEI) of the Northern James Bay Area of Quebec are particularly affected where 21% of adults over the age of 20 are diagnosed as diabetic and 38% as obese [5–10]. This increased prevalence might be caused by their adoption of more westernized way of living: sedentary lifestyle and nontraditional diet (with increased consumption of carbohydrates and saturated fats) in addition to a low-compliance to modern T2D therapies [11–13]. The implementation of educational programs on lifestyle intervention (diet and exercise) has come to no avail, wherefrom the importance to identify alternative and culturally adapted treatments or solutions for obesity and T2D [6, 9–11, 13, 14].

Currently, around 70–95% of the population in the world relies on alternative and complementary medicine in order to respond to their primary healthcare needs. Since the CEI possess a rich traditional pharmacopoeia, we conducted an ethnobotanical survey to identify plant species with potential to treat symptoms related to T2D [15]. These plant species were screened for their antidiabetic potential in extensive *in vitro* studies [16–24]. Of the 17 plants identified, *Larix laricina *K. Koch, belonging to the Pinaceae family, demonstrated antidiabetic potential by increasing glucose uptake and phosphorylation levels of AMPK and ACC in C2C12 myotubes to levels almost comparable to those of metformin [20, 24]. It also potentiated adipogenesis in 3T3-L1 adipocytes, thus acting like the commonly used thiazolidinedione (TZD), rosiglitazone [24]. In addition, we showed that *L. laricina* was one of the strongest uncouplers by severely disrupting mitochondrial function and decreasing ATP production [20]. Uncouplers increase metabolic rate and therefore fuel consumption in order to compensate for decreased ATP, which makes them potential antiobesity agents [20]. Obesity contributes to 55% of all cases of T2D, therefore affecting one or the other might have therapeutic potential for both diseases [25].

There are a variety of medications available on the market, which help in the management of T2D. Some of them, such as the antidiabetic medications metformin (biguanide) and exenatide (GLP-1 analogue), have been reported to produce a minimal amount of weight loss, albeit not strong enough to be considered as an antiobesity agent [26, 27]. The diet-induced obese (DIO) mice are an excellent model to study the well-documented relation of losing weight and improving insulin resistance. It relies on high-calorie diet and inactivity (no genetic involvement) to induce significant weight gain, hyperglycemia, and hyperinsulinemia, thus reflecting the establishment of the metabolic syndrome and a prediabetic state. Therefore, we wanted to study the antidiabetic and antiobesity potential of *L. laricina* in the DIO mouse model using two different administration regiments. Animals either received the plant concomitantly with high fat diet (prevention study) or received the plant after becoming obese and pre-diabetic (treatment study).

## 2. Materials and Methods

### 2.1. Plant Extracts

Specimens of the plant species used in this study, *L. laricina* K. Koch, of the Pinaceae family, were collected in 2004 from the territories of the CEI of Northern Quebec, Canada. Dr. Alain Cuerrier, taxonomist at the Montreal Botanical Garden, confirmed the botanical identity of the plant species. Voucher specimens of the plant species were deposited in the Marie-Victorin Herbarium of the Montreal Botanical Garden in Montreal, QC, Canada (Whap04-11, Mis03-12, Mis03-47). Crude 80% ethanolic extract of *L. laricina* was prepared as previously described [24].

### 2.2. Animals

Four-week-old male nondiabetic C57BL/6 mice were purchased from Charles River Laboratories (Saint-Constant, QC, Canada). All mice had *ad libitum* access to food and water. They were housed in individual cages and maintained on a 12 h light-dark cycle in a temperature-controlled animal room. All experimental protocols were approved by the animal experimentation ethics committee of the Université de Montréal and were carried out in full respect of the guidelines from the Canadian Council for the Care and Protection of Animals.

### 2.3. Prevention Protocol

Following acclimatization, the four-week-old male nondiabetic C57BL/6 mice were divided into four groups of approximately 12 mice each, where they were monitored for 8 weeks. The control groups consisted in administering to one group (Chow) a standard diet purchased from Charles River (18% protein content, 4.5% crude fat, Charles River Animal rodent diet) and to another group (DIO) a high-fat diet (HFD) acquired from Bio-Serv (Bio-Serv Diet no. F3282, Frenchtown, NJ, 60% fat by energy). The remaining groups received the HFD to which was incorporated the dried 80% crude ethanolic plant extract of *L. laricina* at levels adjusted to deliver 125 or 250 mg/kg body weight.

### 2.4. Treatment Protocol

Following acclimatization, the four-week-old male nondiabetic C57BL/6 mice were divided into four groups of approximately 12 mice each. Chow controls received a standard diet (18% protein content, 4.5% crude fat; Charles River Animal rodent diet) for 16 weeks. Other groups were fed a HFD (Bio-Serv Diet no. F3282; 60% energy from fat) for 8 weeks. *L. laricina* at 125 or 250 mg/kg was incorporated in the HFD and treatments continued for an additional 8 weeks, with DIO controls receiving only HFD. Based on published observations and criteria, the animals fed a HFD were segregated into low responders (LRs) and high responders (HRs) (roughly 50/50) according to the data just prior to plant administration (at 8 weeks). Indeed, it has been reported that pooling animals with a more normal metabolic profile even when fed with HFD (such as low weight gain, weak IR, and near-normal glycemia; LR) with animals displaying overt obesity and insulin-resistant state when fed HFD (such as high weight gain, frank IR, and hyperglycemia; HR) can yield misleading results [28]. For simplicity reasons, LR fed with the HFD in the presence or absence of the plant extract will not be depicted since they portrayed an almost normal metabolic profile.

The data representing the effect of a HFD on C57BL/6 mice as compared to their CHOW-fed congeners will not be discussed since the DIO mouse model is well established. However, our model (in both the prevention and treatment studies) follows the expected and published data. In addition, the CHOW group was used as a nonobese control to insure that the model is functional, and therefore the results of this group are not presented. Therefore, the effects of the plant extract, *L. laricina*, will be compared to their respective HFD controls for all the stated parameters, for both the prevention and treatment protocols.

### 2.5. Continuous Physiological and Morphological Parameters

In both protocols body weight, food, and water intake, as well as glycemia, were measured 3 times/week during the course of the study, consistently at the same time, day, in the same order by the same person, throughout the entire duration of the protocols. In order to assess blood glucose levels, blood was collected from puncturing the tail vein and measured using a commercial glucometer (Accu-Check Roche, Montreal, QC, Canada). The area under the curve (AUC) was calculated for these parameters, and the total AUC was then separated into two parts: fraction 1 (F1), representing AUC between weeks 0 and 4 and fraction 2 (F2) corresponding to the AUC between weeks 4 and 8 of plant extract administration. This segregation served to determine the temporal course of action of *L. laricina* in both the prevention and treatment protocols, that is, whether it was effective early in onset (first 4 weeks), later (last 4 weeks), or throughout the study.

### 2.6. Surgical Procedure

At the end of each experimental protocol, the mice were anesthetized using 50 mg/kg pentobarbital intraperitoneally and then sacrificed by exsanguination via the inferior vena cava. During the sacrifice, various organs were removed, collected, and weighed, notably, liver, muscle, white adipose tissue (WAT; epididymal and retroperitoneal fat pads), and subscapular brown fat (BAT). All were placed in liquid nitrogen and then stored at −80°C until further use. As for the livers, they were flushed with a physiological saline solution, weighed, and the median lobes were then dissected, immediately placed in liquid nitrogen, and then stored at −80°C until further use.

### 2.7. Blood Parameters

In order to ensure uninterrupted delivery of plant extract and to avoid the complications of interrupting the dietary plant treatment (for example, drop in food intake and body weight caused by fasting the animals), glycemia, insulin, and adipokines correspond to nonfasting measurements. Plasma insulin, adiponectin, and leptin were assessed by radioimmunoassay (RIA: Linco Research, St. Charles, MO, USA) according to manufacturer's instructions.

### 2.8. Tissue Triglyceride Measurement

Tissue triglyceride content was measured by grinding up into powder, under liquid nitrogen, around 100 mg of each of the collected liver and muscle samples, and then using Folch's chloroform/methanol (2 : 1) extraction method [29]. Triglyceride content was then quantified using a commercial kit (Randox Laboratories Ltd., UK).

### 2.9. Skin Temperature

In the prevention protocol, after 4 and 8 weeks of treatment, the temperature of the animals was read and recorded with a digital thermometer (Cole-Parmer Instrument Company, USA) by placing a probe on the external intercostal region of the animal for 2 minutes. This procedure is noninvasive and the least stressful for the animals [30].

### 2.10. Isolation of Mitochondria and Measurement of Respiration

Following anesthetization, the livers of mice from the treatment protocol were flushed with the Krebs-Henseleit buffer (pH 7.4, 22°C). Mitochondria were then isolated following the method of Johnson and Lardy, as previously described [31–33].

### 2.11. Statistical Analysis

Data were analyzed by one-way analysis of variance (ANOVA), followed by post hoc analysis (Bonferroni-Dunn test or Holm-Sidak) as appropriate using Sigma Stat software (Jandel Scientific, San Rafael, CA, USA). Areas under the curve (AUC) were calculated by using PRISM software (GraphPad, San Diego, CA, USA). Data are expressed as mean ± SEM of the indicated number of determinations. Statistical significance was set at  *P* < 0.05.

## 3. Results

### 3.1. *L. laricina* Significantly Improved Glycemia in the Treatment Protocol

**Figure 1:**
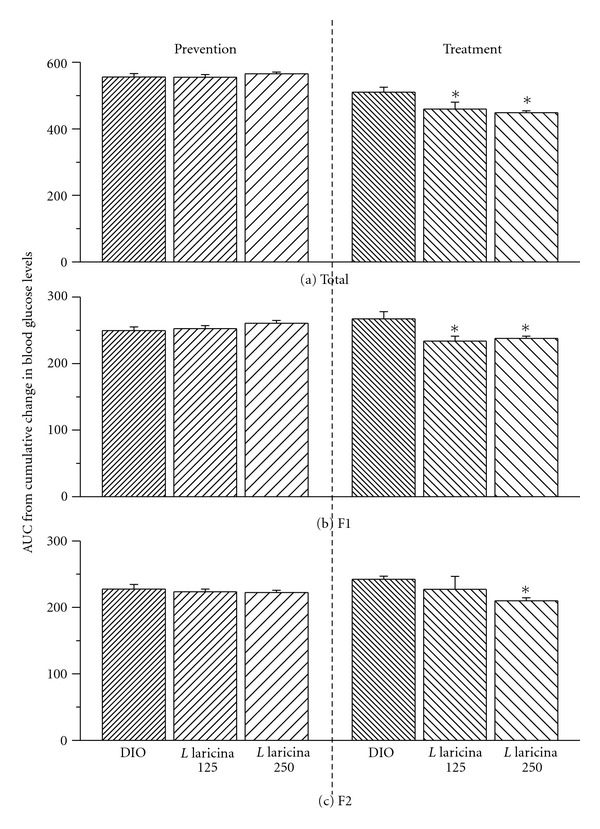
Area under the curve (AUC) of nonfasting glycemia levels, in C57BL/6 mice treated with either HFD (DIO) or *L. laricina* at 125 or 250 mg/kg, which was incorporated in the HFD for 8 weeks in the prevention protocol and for the last 8 of 16 weeks in the treatment protocol. Total AUC for blood glucose levels versus time (a) was calculated and then fractionated into the first and second half of the feeding period corresponding to weeks 0–4 (AUC_F1_; b) and weeks 4–8 (AUC_F2_; c), respectively. All values are mean ± SEM. Number of animals/group for prevention protocol DIO = 11; *L. laricina* 125 = 13; *L. laricina* 250 = 13, and for the treatment protocol DIO = 7; *L. laricina* 125 = 5; *L. laricina* 250 = 8). *denotes significantly different as compared to DIO group (one-way ANOVA; post hoc analysis Holm-Sidak or Bonferroni-Dunn test; *P* < 0.05).

Glycemia levels, which increased with HFD as compared to CHOW (by 19%; data not shown), were not significantly altered when *L. laricina* was added concomitantly with the HFD (prevention study; NS; Figures 1(a)–1(c)). However, HFD-induced hyperglycemia (32% as compared to CHOW congeners; data not shown) significantly decreased in the group receiving the plant as treatment (following 8 weeks on HFD; Figures 1(a)–1(c)). Indeed, blood glucose levels, as measured by the area under the curve (AUC) of glycemia versus time, decreased in a dose-dependent manner by 10% and 12% with 125 mg/kg and 250 mg/kg doses, respectively (AUC_T_; *P* < 0.05; Figure 1(a)). In order to determine the temporal aspect of this antihyperglycemic effect, we fractionated the AUC between the first month of treatment (weeks 0–4: AUC_F1_; Figure 1(b)) and second month (weeks 4–8: AUC_F2_; Figure 1(c)). Our findings show that *L. laricina* lowered glycemia levels from the onset of the treatment (by 11–13%; *P* < 0.05; Figure 1(b)), and this was maintained throughout the treatment, albeit remained significant with highest dose only (13% at 250 mg/kg; *P* < 0.05; Figure 1(c)).

### 3.2. *L. laricina* Significantly Decreased Insulin Levels in the Treatment Protocol Only, While Decreasing Leptin/Adiponectin Ratio in Both Protocols

Administration of either dose of *L. laricina* tended to lower insulin levels by 25% to 35% in the prevention study, but failed to reach statistical significance (NS; Table 1). In the treatment protocol, however, *L. laricina-*induced decrease of insulinemia levels reached 72% with the 250 mg/kg dose (*P* < 0.05; Table 1), suggesting improvement of insulin resistance state and coinciding with the plant's highest effect on glycemia as mentioned above.

Other indicators were measured to confirm the re-establishment of insulin sensitivity. Adipose tissue is considered an endocrine organ, releasing into circulation adipokines, such as leptin and adiponectin, involved in the development of insulin resistance. A decrease of leptin/adiponectin ratio is thus considered as a marker of improved insulin sensitivity. In both prevention and treatment protocols, *L. laricina* tended to increase adiponectin levels by 16–26%, however, reaching statistical significance with the 125 mg/kg dose in the prevention protocol only (*P* < 0.05; Table 1). In parallel, leptin levels were reduced with *L. laricina* administration by 4–16% in the prevention study and by 21–30% in the treatment study, without, however, being statistically significant (NS; Table 1). Overall, these changes in adipokines levels resulted in significant decrease of the leptin/adiponectin ratio. Indeed, administration of *L. laricina* significantly lowered this ratio by 37% at 250 mg/kg in the prevention study (*P* < 0.05; Table 1) and by 29–31% with both doses in the treatment study (*P* < 0.05; Table 1). Therefore, *L. laricina* seems to decrease systemic insulin resistance.

Furthermore, since accumulation of lipids in the liver and skeletal muscle have been implicated in insulin resistance, we measured hepatic and muscular triglyceride (TG) levels. Despite *L. laricina* decreasing systemic insulin resistance, as suggested by improvement in the aforementioned parameters, hepatic or muscular triglyceride levels were not significantly altered (NS, Table 2).

### 3.3. *L. laricina* Diminished Body Weight Gain in Both the Prevention and Treatment Protocols, While Decreasing Fat Pad Weight in the Prevention Protocol Only

**Figure 2:**
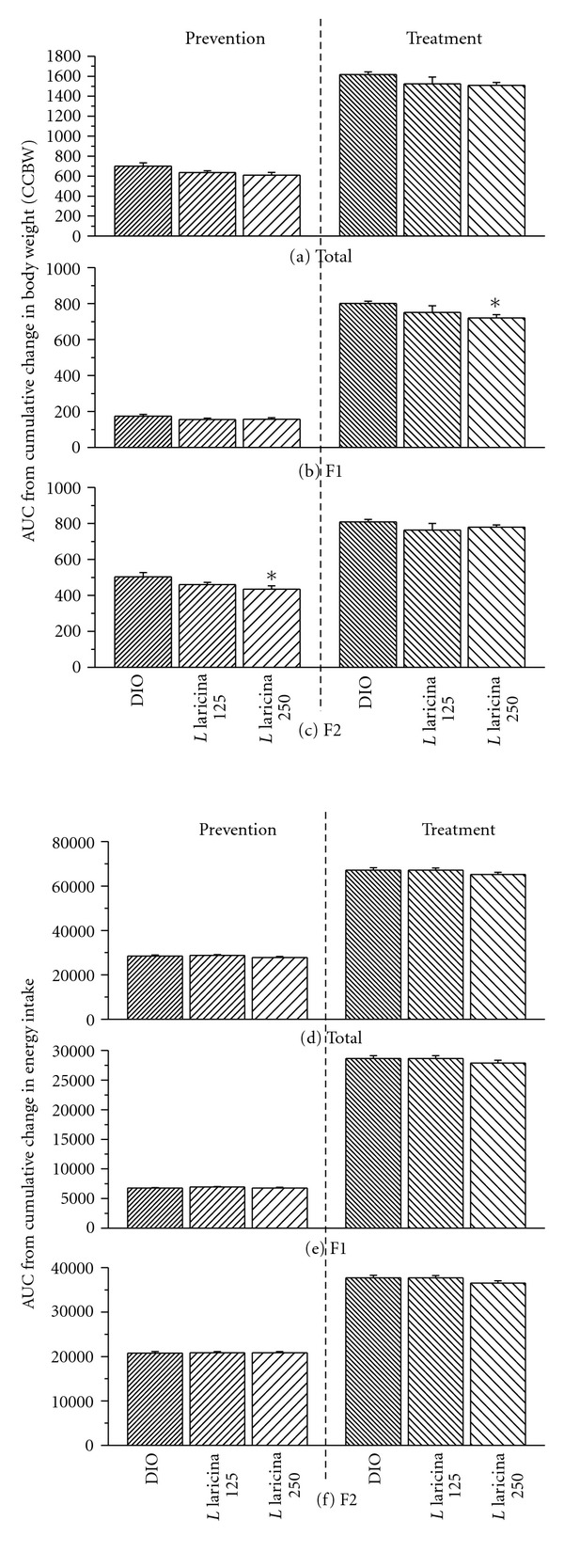
Area under the curve (AUC) of cumulative change in body weight (CCBW; (a–c)) and cumulative change in energy intake (CCEI; d–f) in C57BL/6 mice treated with either HFD (DIO), or *L. laricina* at 125 or 250 mg/kg, which was incorporated in the HFD for 8 weeks in the prevention protocol and for the last 8 of 16 weeks in the treatment protocol. Total AUC for CCBW-versus-time (a) or CCEI-versus-time (d) was calculated and then fractionated into the first and second half of the feeding period corresponding to weeks 0–4 (AUC_F1_; b or e) and weeks 4–8 (AUC_F2_; c or f), respectively. All values are mean ± SEM. Number of animals/group for prevention protocol DIO = 11; *L. laricina* 125 = 13; *L. laricina* 250 = 13, and for the treatment protocol DIO = 7; *L. laricina* 125 = 5; *L. laricina* 250 = 8). *denotes significantly different as compared to DIO group (one way ANOVA; post-hoc analysis Holm-Sidak or Bonferroni-Dunn test; *P* < 0.05).

Continuous measurements of cumulative change in body weight (CCBW), represented as the area under the curve (AUC), revealed that while the effect of *L. laricina* on body weight gain was immediate in the prevention protocol for both doses (10% for AUC_F1_; NS; Figure 2(b)), it only reached significant proportions in the second half of the protocol (AUC_F2_) with the highest dose, decreasing it by 14% as compared to DIO controls (AUC_F2_; *P* < 0.05; Figure 2(c)). In contrast, in the treatment protocol, *L. laricina* produced its strongest and most significant effect in the first half of administration lowering AUC_F1_-CCBW by 10% at 250 mg/kg as compared to DIO congeners (AUC_F1_; *P* < 0.05; Figure 2(b)). However, its antiobesity effect was not maintained; *L. laricina* reduced AUC_F2_ by only 4% at 250 mg/kg (NS; Figure 2(c)).

Consistent with the observed decrease in body weight gain, *L. laricina* significantly lowered retroperitoneal/abdominal fat pad weight in the prevention study by 15% at 250 mg/kg (*P* < 0.05; Table 3) as compared to DIO controls. As for the treatment, it corresponded to a slight decrease with both doses (5%–11%; NS; Table 3).

It is interesting to note that, in both protocols, mice administered *L. laricina* maintained similar food intake to their DIO controls (NS; Figures 2(d)–2(f)), while being less prone to gaining weight. Finally, *L. laricina* exhibited no toxicity as demonstrated by unaltered blood biochemical parameters and tissue histological examination (data not shown).

### 3.4. *L. laricina* Improved Mitochondrial Function

**Figure 3:**
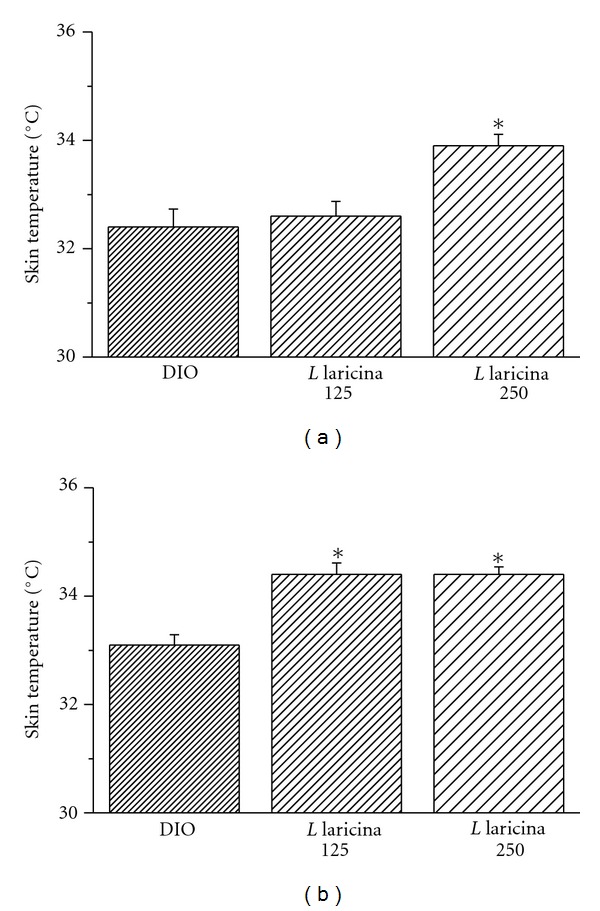
Skin temperature was measured in the prevention study after 4 weeks (a) and after 8 weeks (b) of treatment, from mice treated with either HFD (DIO) or *L. laricina* at 125 or 250 mg/kg, which was incorporated in the HFD for 8 weeks. All values are mean ± SEM. Number of animals/group for prevention protocol DIO = 11; *L. laricina* 125 = 13; *L. laricina* 250 = 13. *denotes significantly different as compared to DIO group (one-way ANOVA; post hoc analysis Holm-Sidak or Bonferroni-Dunn test; *P* < 0.05).

Regulation of body temperature requires regulating both heat production and heat loss. Mitochondria metabolism is an important source of heat production. For the most part, variations in the rate of electron transport are directly related to the demand by the cells for ATP. However, exogenous substances, which uncouple mitochondria, can lead to the disruption of oxidative phosphorylation, decreasing ATP synthesis and increasing heat production. Previous screening studies have shown that *L. laricina* uncoupled mitochondrial function in isolated Wistar rat hepatocytes [20]. Therefore, we used skin temperature as an indirect measure of energy expenditure and possible mitochondrial uncoupling in the prevention study. After 4 and 8 weeks of treatment, we observed a gradual and dose-dependent increase in skin temperature with *L. laricina* administration (*P* < 0.05; Figures 3(a) and 3(b)).

 Having perfected the isolation of mitochondria in mice, we opted to directly evaluate mitochondrial function in the treatment study in plant-treated mice compared to DIO controls. As expected DIO mice, which have increased fatty acid deposition in the liver, exhibited a lower respiratory control ratio (RCR) accompanied by decreased ATP production, in comparison to CHOW animals, although data variability precluded statistical significance (Table 4). Despite a small sample, animals treated with 250 mg/kg of *L. laricina* seemed to restore mitochondrial function and capacity to the level of chow values, as observed by an increase in RCR and ATP synthesis (Table 4).

## 4. Discussion

According to the International Diabetes Federation latest figures, the number of people living with diabetes will rise from 366 million in 2011 to 552 millions by 2030 [34]. The magnitude and impact of this disease dictate the urgent need for action. Although several drugs exist on the market to treat diabetes, the need to discover novel therapeutic options is warranted, especially in aboriginal context, such as the CEI [6, 9–11, 13, 14]. Indeed, recent lifestyle changes and noncompliance with western medicines could account for the staggering diabetes prevalence of 29% in adults over 20 years old in this community [5, 6, 11–13].

In our quest to discover a culturally adapted diabetes treatment, we identified *L. laricina* from the CEI pharmacopoeia as potential antidiabetic medicinal plant. Initial *in vitro* screening showed that this plant stimulated glucose uptake, potentiated adipogenesis [24], activated AMPK, and acted as mitochondrial uncoupler/inhibitor (on normal isolated mitochondria) [20]. These effects are reminiscent of the action of Metformin, which partially mediates its action through inhibition of mitochondrial respiration, activation of AMPK, and upregulation of glucose uptake as well [20, 35–39]. It was therefore interesting to test the antidiabetic effect of *L. laricina* in a mouse model mimicking type 2 diabetes as a consequence of obesity by subjecting C57BL/6 mice to HFD (diet-induced-obesity model; DIO). We tested the plant in two different regiments: (1) *L. laricina* was administered concomitantly with HFD for 8 weeks in order to confirm its capacity to attenuate the development of obesity, diabetes, and the associated insulin-resistance (prevention study); (2) *L. laricina* was administered to obese and insulin resistant C57BL/6 mice (already on HFD for 8 weeks) for 8 weeks to test its ability to reverse the establishment of both of these states (treatment study).

Typically, the DIO model is characterized by increased body weight gain, hyperglycemia, and establishment of insulin-resistant state (hyperinsulinemia, increase of the leptin/adiponectin ratio, ectopic fat storage in the liver and muscle) [40, 41]. We therefore examined these parameters to determine the effect of *L. laricina in vivo.*

 The results of the present studies confirm that *L. laricina* holds great promise as an antidiabetic medicinal plant. Although this plant had no effect of glycemia when administered concomitantly with HFD (prevention study), on the contrary, it significantly and dose-dependently decreased glycemia in the treatment study. These findings correlate well with our *in vitro* data where this plant extract increased glucose uptake in skeletal muscle cells and adipocytes [24], which accounts for 85% of postprandial glucose disposal, [42] and increased AMPK activity in C2C12 muscle cells [20]. It is worthy to note that glycemia levels of animals receiving HFD in the treatment study are higher than those in the prevention study (32% versus 19%, respectively, compared to CHOW). One could suggest that *L. laricina* exerts its antihyperglycemic effect better when disease processes are more pronounced, thus explaining the observed difference in the plant's effect between the prevention and the treatment study.

Insulin resistance parameters were also modulated with administration of *L. laricina* in both treatment regiments. While strong tendencies are apparent in the prevention study, insulinemia and leptin/adiponectin ratio were significantly decreased in the treatment study (especially with the highest dose of *L. laricina*), suggesting improvement of systemic insulin resistance. Intriguingly, *L. laricina* failed to decrease hepatic and muscle triglycerides in both studies. Several lines of evidence suggest that hepatic triglyceride accumulation leads to insulin-defective signaling in the liver with increased hepatic glucose output. However, Buettner et al. have shown that TG accumulation in the liver is not always sufficient to impair insulin signaling [43, 44]. In fact, they argue that systemic factors (such as adipokines, free fatty acids, pro-inflammatory cytokines) may play an important role in the regulation of hepatic glucose output and insulin sensitivity *in vivo* [43, 44]. Hence, our data on the lack of depletion in intrahepatic and intramuscular triglyceride levels needs to be evaluated in further detail. Indeed, continued administration of HFD alongside *L. laricina* could make elimination of steatosis difficult. Another possibility could be that since oxidation pathways are saturated with fatty acids being mobilized from the adipose tissue (decrease in adipose tissue weight due to probable hormone-sensitive lipase activity), this could consequently hinder any decrease in tissue triglyceride stores [45]. In all cases, since adiponectin levels tended to increase and leptin/adiponectin ratio (an indicator of insulin resistance) [46–50] significantly decreased with ingestion of *L. laricina*, proinsulin-resistant systemic factors seem to be decreased and insulin sensitivity improved. Interestingly, we have shown that treatment of hepatic cells *in vitro*  by *L. laricina* inhibits the activity of enzymes implicated in hepatic gluconeogenesis, such as glucose-6-phosphatase and activates those promoting glycogen formation, such as glycogen synthase (GS), thus directly modulating hepatic glucose output [51].

This plant showed slight decrease of body weight with both studies, which was significant if continuous measurements were taken into account for the first and second month of administration. These changes occurred while the animals were on a continuous hypercaloric/fat-laden diet and without any observed change in energy intake. This could represent an indirect modulation of body weight as a consequence of *L. laricina* antidiabetic activity, which in some cases is similar to Metformin.

*L. laricina* administration also decreased retroperitoneal fat pad weight significantly in the prevention study and showed a tendency to do so in the treatment study. This represents an important action in the fight against insulin resistance since visceral adipose tissue has been implicated in the detrimental effects of obesity and insulin resistance [52]. Hence, modulation of this tissue would influence adipokine secretion and contribute to the improvement of insulin sensitivity, as can be seen in our plant-treated mice.

On the molecular level, we have shown that *L. laricina* activates AMPK in C2C12 myotubes [20] and H4IIE hepatic cell line [51]. This activation may be secondary to a variety of factors, including adiponectin or metabolic stress induced by the disruption of mitochondrial energy transduction [53–56]. In the literature, it has been reported that animals (mice or rat) fed a high-fat diet exhibit a decreased mitochondrial respiratory capacity (state 3/state 4), as was observed in this treatment protocol in mice administered a HFD [57]. Increased consumption of dietary fat may lead to alterations in mitochondrial membrane composition and increased ROS production and peroxidation of fatty acids, which could damage mitochondrial structures, all affecting mitochondrial function [57]. Uncoupling agents are beneficial in alleviating the mitochondrial stress induced by a HFD, by increasing fatty acid oxidation and decreasing ROS production [58]. In the treatment study, *L. laricina* at 250 mg/kg improved mitochondrial capacity and ATP production to levels comparable to those observed in animals fed a standard Chow diet. As demonstrated in previous *in vitro* screening studies, the uncoupling effect of *L. laricina* is short-lived and is followed by a prolonged activation of AMPK and an overshoot phenomenon occurring to restore energy homeostasis, where ATP production is greatly increased, through raised carbohydrate and lipid oxidation [20]. Other benefits of increased AMPK activity include protecting cells from further damage by potentiating mitochondrial biogenesis [20, 59, 60]. Therefore, it seems that in the current animal treatment protocol, the long-term effect of *L. laricina* improved mitochondrial capacity and most probably through AMPK activation regulated glucose homeostasis. Of note, uncoupling agents usually lead to increased heat production due to increased energy expenditure. *L. laricina-*treated animals in the prevention study exhibited elevated skin temperature, thus confirming its uncoupling activity* in vivo*.

In conclusion, this study confirms the antidiabetic activity of *L. laricina* in the context of diet-induced obesity in a mouse model. The results clearly show that *L. laricina* decreased hyperglycemia and insulin resistance and improved mitochondrial function in the treatment study, while partially modulating parameters involved in insulin sensitivity in the prevention one. It also had a slight effect on body weight gain in both studies. The exact mechanisms of action of *L. laricina* remain to be identified, but results point toward possible activation of AMPK and its downstream effectors. In view of the soaring increase in both obesity and diabetes among aboriginal populations and in particular the CEI, *L. laricina* represents a valuable alternative, and culturally adapted treatment for both these diseases.

## Figures and Tables

**Table 1:** Effects of HFD and *L. laricina* administration on systemic parameters at sacrifice.

	Prevention protocol			Treatment protocol		
	DIO	*L. laricina*	*L. laricina*	DIO	*L. laricina*	*L. laricina*
		125 mg/kg	250 mg/kg		125 mg/kg	250 mg/kg
Insulin (ng/mL)	9.2 ± 1.8	6.9 ± 1.1	6.0 ± 0.9	39.9 ± 5.8	29.4 ± 9.1	11.0 ± 1.9*
Leptin (ng/mL)	39.3 ± 3.5	37.7 ± 3.2	33.1 ± 2.0	39.0 ± 3.0	27.1 ± 5.4	30.9 ± 2.1
Adiponectin (*μ*g/mL)	8.8 ± 0.7	11.1 ± 0.8*	10.8 ± 0.4	11.1 ± 0.6	10.9 ± 0.7	12.8 ± 0.6
Leptin/adiponectin ratio	4.9 ± 0.6	3.7 ± 0.5	3.1 ± 0.2*	3.5 ± 0.2	2.5 ± 0.5*	2.4 ± 0.2*

Measurements were obtained after 8 weeks (prevention) or 16 weeks (treatment) of administration with either HFD (DIO) or *L. laricina* at 125 or 250 mg/kg, which was incorporated in the HFD for 8 weeks in the prevention protocol and for the last 8 of 16 weeks in the treatment protocol. All values represent the mean ± SEM (prevention protocol DIO = 11; *L. laricina* 125 = 13; *L. laricina* 250 = 13, and for the treatment protocol DIO = 7; *L. laricina* 125 = 5; *L. laricina* 250 = 8). ^∗^denotes that treated groups are significantly different as compared to DIO (one-way ANOVA; post hoc analysis Holm-Sidak or Bonferroni-Dunn test; *P* < 0.05).

**Table 2:** Effects of HFD and *L. laricina* administration on hepatic and muscular triglyceride accumulation.

	Prevention protocol			Treatment protocol		
	DIO	*L. laricina*	*L. laricina*	DIO	*L. laricina*	*L. laricina*
		125 mg/kg	250 mg/kg		125 mg/kg	250 mg/kg
Liver TG levels (mg/g total liver)	331 ± 54	407 ± 47	374 ± 52	1041 ± 173	919 ± 240	1138 ± 118
Muscle TG levels (*μ*g/mg)	84 ± 12	60 ± 6	65 ± 8	212 ± 29	224 ± 80	255 ± 33

The colorimetric dosage of TG levels in both the liver and muscle was determined using a commercial kit (as described in detail in Section 2.8). Measurements were obtained after 8 (prevention) or 16 (treatment) weeks of administration with either HFD (DIO) or *L. laricina* at 125 or 250 mg/kg, which was incorporated in the HFD for 8 weeks in the prevention protocol and for the last 8 of 16 weeks in the treatment protocol. All values represent the mean ± SEM (prevention protocol DIO = 11; *L. laricina* 125 = 13; *L. laricina* 250 = 13, and for the treatment protocol DIO = 7; *L. laricina* 125 = 5; *L. laricina* 250 = 8).

**Table 3:** Effects of HFD and *L. laricina* administration on organ weights at sacrifice.

	Prevention protocol			Treatment protocol		
	DIO	*L. laricina*	*L. laricina*	DIO	*L. laricina*	*L. laricina*
		125 mg/kg	250 mg/kg		125 mg/kg	250 mg/kg
Retroperitoneal fat pad (g)	1.34 ± 0.05	1.26 ± 0.04	1.14 ± 0.05*	1.51 ± 0.08	1.35 ± 0.24	1.44 ± 0.04
Epididymal fat pad (g)	2.40 ± 1.00	2.56 ± 0.08	2.56 ± 0.07	1.21 ± 0.03	1.32 ± 0.18	1.85 ± 0.12*
Brown fat pad (g)	0.30 ± 0.03	0.33 ± 0.01	0.28 ± 0.02	0.44 ± 0.01	0.37 ± 0.06	0.42 ± 0.02
Liver weight (g)	1.77 ± 0.09	1.85 ± 0.05	1.80 ± 0.07	2.62 ± 0.15	2.44 ± 0.38	2.57 ± 0.12
Liver index	410 ± 2	429 ± 1	437 ± 1	551 ± 3	539 ± 5	555 ± 2

Measurements were obtained after 8 weeks (prevention) or 16 weeks (treatment) of administration with either HFD (DIO) or *L. laricina* at 125 or 250 mg/kg, which was incorporated in the HFD for 8 weeks in the prevention protocol and for the last 8 of 16 weeks in the treatment protocol. The liver index corresponds to liver weight (mg)/body weight (mg). All values represent the mean ± SEM (prevention protocol DIO = 11; *L. laricina* 125 = 13; *L. laricina* 250 = 13, and for the treatment protocol DIO = 7; *L. laricina* 125 = 5; *L. laricina* 250 = 8). ^∗^denotes that treated groups are significantly different as compared to DIO (one-way ANOVA; post hoc analysis Holm-Sidak or Bonferroni-Dunn test; *P* < 0.05).

**Table 4:** Effects of obesity as well as *L. laricina* administration on hepatic mitochondrial function.

	Treatment protocol			
	Chow	DIO	*L. laricina*	*L. laricina*
			125 mg/kg	250 mg/kg
State 3	62.90 ± 4.50	59.81 ± 8.34	51.41 ± 6.91	62.91 ± 3.34
State 4	18.23 ± 1.05	17.43 ± 0.89	16.20 ± 1.04	16.29 ± 0.85
RCR	3.45 ± 0.08	3.37 ± 0.32	3.18 ± 0.35	3.87 ± 0.14
ATP synthesis	3.53 ± 0.53	3.20 ± 0.52	2.73 ± 0.40	3.52 ± 0.25

Mitochondrial function was measured as described in detail in Section 2.10, after 16 weeks of administration with either standard diet (CHOW), HFD (DIO), or *L. laricina* at 125 or 250 mg/kg, which was incorporated in the last 8 of 16 weeks in the treatment protocol. State 3 represents the rate of oxygen consumed during oxidative phosphorylation; state 4 represents the rate of oxygen consumption obtained after oxidative phosphorylation; RCR (respiratory control ratio) represents the ratio between state 3 and state 4. All values represent the mean ± SEM (for the treatment protocol CHOW = 4; DIO = 5; *L. laricina* 125 = 4*; L. laricina* 250 = 4).
